# Lignin-Modified Carbon Nanotube/Graphene Hybrid Coating as Efficient Flame Retardant

**DOI:** 10.3390/ijms18112368

**Published:** 2017-11-08

**Authors:** Kunlin Song, Indroneil Ganguly, Ivan Eastin, Anthony B. Dichiara

**Affiliations:** 1 School of Environmental and Forest Sciences (SEFS), University of Washington, 4000 15th Ave NE, Seattle, WA 98195, USA; ksong2@uw.edu; 2 Center of International Trade in Forest Products (CINTRAFOR), School of Environmental and Forest Sciences (SEFS), University of Washington, 4000 15th Ave NE, Seattle, WA 98195, USA; indro@uw.edu (I.G.); eastin@uw.edu (I.E.)

**Keywords:** lignin, cellulose paper, flame retardancy, carbon nanomaterials

## Abstract

To reduce fire hazards and expand high-value applications of lignocellulosic materials, thin films comprising graphene nanoplatelets (GnPs) and multi-wall carbon nanotubes (CNTs) pre-adsorbed with alkali lignin were deposited by a Meyer rod process. Lightweight and highly flexible papers with increased gas impermeability were obtained by coating a protective layer of carbon nanomaterials in a randomly oriented and overlapped network structure. Assessment of the thermal and flammability properties of papers containing as low as 4 wt % carbon nanomaterials exhibited self-extinguishing behavior and yielded up to 83.5% and 87.7% reduction in weight loss and burning area, respectively, compared to the blank papers. The maximum burning temperature as measured by infrared pyrometry also decreased from 834 °C to 705 °C with the presence of flame retardants. Furthermore, papers coated with composites of GnPs and CNTs pre-adsorbed with lignin showed enhanced thermal stability and superior fire resistance than samples treated with either component alone. These outstanding flame-retardant properties can be attributed to the synergistic effects between GnPs, CNTs and lignin, enhancing physical barrier characteristics, formation of char and thermal management of the material. These results provide great opportunities for the development of efficient, cost-effective and environmentally sustainable flame retardants.

## 1. Introduction

The use of fire as a source of energy has been critical for the development of human civilization. However, fire hazards also present serious threats to people’s life and property, and have recently attracted considerable attention from governments and society. In 2015, USA fire departments responded to an estimated 1,345,500 fires, causing 3280 civilian fire fatalities, 15,700 injuries and an estimated $14.3 billion in direct property loss [1]. Most materials used in building and construction can be rapidly oxidized in the exothermic combustion process in the presence of oxygen and heat. Cellulose-based materials, in particular, are easily ignitable and susceptible to degradation at elevated temperatures, and require the addition of flame retardants to improve their resistance to fire [2,3,4]. Flame retardants typically contain bromine, chlorine, phosphorus, nitrogen, metals, or minerals based on aluminum and magnesium [5,6]. For instance, ammonium polyphosphate [7,8], diammonium phosphate [9], hydroxyapatite (Ca_10_(OH)_2_(PO_4_)_6_) [10], poly(vinylphosphonic acid) [11] and poly(methylenephosphine) [12] have been employed to endow cellulose-based materials (e.g., pulp fibers, paper and cotton fabrics) with fire-resistance properties. Nevertheless, conventional flame retardants, especially halogenated compounds, require relatively large quantities to be efficient, have low tolerance to chemicals such as oxidants and organic solvents, and exhibit negative environmental and health impacts. Therefore, the development of flame retardants with good chemical stability and environmentally sustainable characteristics is critical to improve safety awareness of consumers and meet increasing fire resistance requirements.

Recently, carbon nanomaterials such as graphene nanoplatelets (GnPs) and carbon nanotubes (CNTs) have drawn tremendous attention due to their superior flame-retarding properties in polymer composites, including polypropylene [13,14], epoxy [15], poly(vinyl alcohol) [16] and poly(methyl methacrylate) [17]. The incorporation of these carbon nanoparticles significantly delayed ignition and reduced the heat-release rate during the combustion of polymer composites. Moreover, they provided other essential properties for enhanced fire protection, such as char formation [18], smoke and toxic gas suppression [19], and physical barriers to oxygen and gaseous pyrolysis products [20]. However, the application of carbon nanomaterials as flame-retarding agents for cellulose-rich substrates so far is scant. Papers prepared from bleached hardwood Kraft pulp comprising 35 wt % CNTs produced a lower effective heat of combustion than the blank samples [21]. Other flame-retardant papers were made by incorporating graphene oxide into the aqueous pulp, and demonstrated good flame retardancy [18]. To improve fire protection further, surface coating is typically considered as a convenient, economical and efficient approach, due to its advantages including easy accumulation of flame retardants at the substrate surface forming a protective layer, and simultaneously preserving the bulk properties of the substrate [22]. For instance, graphene phosphonic acid composite was deposited as a fire-protective layer on the surface of papers by dip coating with a loading amount of 13.6 wt % [23]. Pine sawdust was also soaked into a suspension of reduced graphene oxide and sodium metaborate hydrates with a loading of 13.4% to improve its flame retardancy [24]. These reports have shown great promise as effective flame retardants; nevertheless, opportunities exist for improved fire resistance, especially at low nanomaterial content (i.e., <10 wt %). Reducing the loading of carbon nanoparticles is of paramount importance to minimize environmental impact and ensure that the price of the resulting materials would not increase significantly compared to that of untreated ones.

The present research describes the preparation of aqueous mixtures of GnPs, CNTs and lignin as environmentally sustainable precursors for the deposition of a fire-protective layer on the surface of papers by Meyer rod coating. Compared to other methods, the Meyer rod coating is a reproducible technique for the continuous large-scale preparation of uniform carbon nanomaterial films, which is critical for the formation of a protective barrier to limit soot transfer [25]. Furthermore, the combination of GnPs with CNTs in the correct proportions has the potential to yield synergistic effects [26,27], while the incorporation of lignin, the second most abundant natural polymer typically treated as a waste byproduct of the pulp and paper industry, can ensure high dispersion quality of nanomaterials [28], and provide good char-forming ability [29,30,31]. Considering the above, we successfully developed a cost-effective, high-performance and environmentally sustainable composite flame retardant with nanomaterial content as low as 4 wt %. The surface morphology, gas permeability, chemical structure and fire resistance of the as-prepared flame-retardant papers were thoroughly studied. The effects of coating content and formulation—with different ratios of GnPs, CNTs and lignin—on the fire-retardant properties were examined, and the flame-retardant mechanism was discussed.

## 2. Results and Discussion

### 2.1. Characterization of Coated Papers

**Figure 1:**
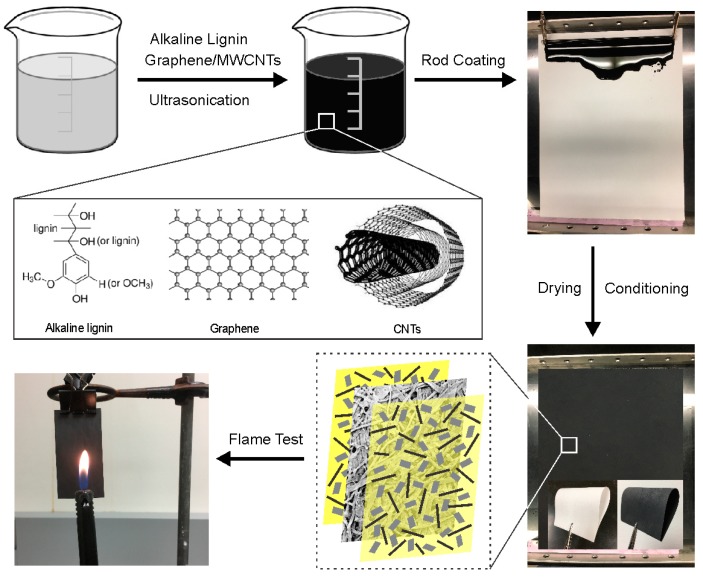
The schematic showing the fabrication and flame test of the GnP/CNT/lignin-coated papers.

Figure 1 illustrates the fabrication process of the fire-resistant papers. The rod-coating method was used to precisely control the thickness of the protective layer by adjusting the volume and solution concentration of the precursors. The average thickness of the plain paper was 113 μm and reached 164–169 μm after coating with 2.5 g/m^2^ of carbon nanomaterials (Table 1). Notably, no obvious differences in thickness were observed among the papers coated with different combinations of carbon nanomaterials at the same loading amount.

**Figure 2:**
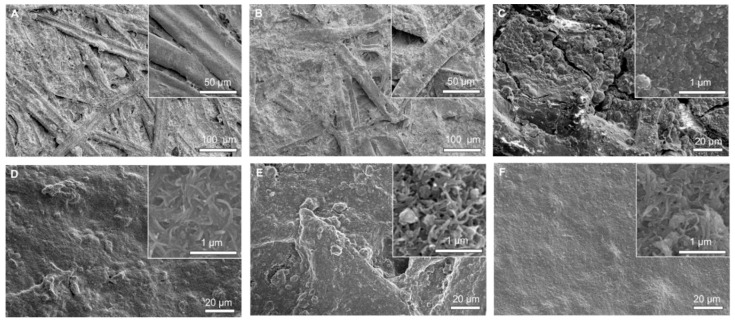
Scanning electron microscope (SEM) images of the paper coated by different carbon materials (**A**): control paper; (**B**): lignin-coated paper; (**C**): G-2.5; (**D**): M-2.5; (**E**): GM-2.5; (**F**): GM0.5-2.5.

When the GnP:CNT ratio was kept at 1:2 and the content of carbon nanomaterials increased from 1.7 to 5.8 g/m^2^, the thickness of the resulting papers increased accordingly from 166 μm (GM0.5-1.7) to 188 μm (GM0.5-5.8). Variations in thickness across 15 different locations per sheet remained lower than 2% for all specimens, revealing the good uniformity of the coating process, which was further demonstrated by scanning electron microscope (SEM) observations illustrated in Figure 2. In the plain paper without coating, individual cellulose microfibers with different sizes were nested together creating a cellulose matrix (Figure 2A). When lignin was used as the sole coating material (Figure 2B), some cellulose nanofibers were still visibly exposed, meaning that lignin did not fully cover the whole paper surface. In all other cases, the absence of visible aggregates yielded relatively flat carbon layers of 25–30 μm in thickness on the paper surface regardless of the type of materials used for coating (Figure 2C–F), confirming the good dispersion of carbon nanomaterials during the process [28,32]. This can be attributed to the strong capillary forces of the porous paper, and the resulting large contact area between the coated materials and the cellulose microfibers. The addition of lignin with numerous hydrophilic groups also promoted the adsorption of carbon nanomaterials on the surface of hydrophilic cellulose fibers. The surface texture, however, was found to vary with the nature of the thin films. Except for the GnP-coated samples, where obvious micro-cracks were observed (Figure 2C), all specimens exhibited a relatively smooth surface. One-dimensional CNTs formed an entangled network consisting of randomly aligned CNT bundles with macropores of diameter up to 400 nm (Figure 2D). This was consistent with pore analysis of similar CNT membranes by mercury intrusion porosimetry, showing a broad size distribution with peak pore size of several hundred nm [33]. Interestingly, the apparent smoothness of the hybrid coating comprising both GnPs and CNTs at a mass ratio of 1:2 was much better than that of either component alone, with the larger pores in the CNT network being filled by the two-dimensional GnPs (Figure 2F). It is also worth noting that the surface smoothness decreased when higher amounts of GnPs were added in the hybrid mixture (Figure 2E). Therefore, the hierarchical GnP/CNT hybrid structure was expected to have better barrier effect and flame retardant performance than those using either component alone.

**Figure 3:**
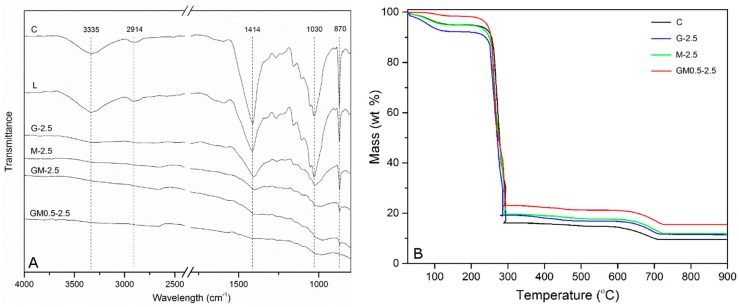
Representative Fourier transformed infrared (FTIR) spectra (**A**) and thermograms (**B**) of the blank and coated papers.

The Fourier transformed infrared (FTIR) spectra of the different coated papers are shown in Figure 3A. The distinctive infrared (IR) peaks of cellulose were present in the plain paper (i.e., no coating), including O–H stretching at 3335 cm^−1^, CH_2_ asymmetrical stretching at 2914 cm^−1^, CH_2_ scissoring at 1414 cm^−1^, C–O stretching at 1030 cm^−1^ and C–H out-of-plane bending at 870 cm^−1^ [34,35,36]. After coating, the intensities of the characteristic peaks of cellulose listed above gradually decreased depending on the coating composition, in the order of L, G-2.5, M-2.5, GM-2.5 and GM0.5-2.5. It is well known that the IR light passes through the attenuated total reflection (ATR) crystal and penetrates into the sample contacting the crystal with a depth of several micrometers, which is in the range of the different coating thicknesses reported in this work (i.e., 25–30 μm). Meanwhile, carbon nanomaterials, such as GnPs and CNTs, typically showed very strong absorption in the IR region. Therefore, it is reasonable to assume that the IR-active groups of the cellulose substrate covered by coatings tens of micrometers thick would not be detected unless being directly exposed to the IR light due to the presence of micro-cracks or poorly coated regions. FTIR spectroscopy with ATR attachment can thus be utilized as a way to investigate the density and uniformity of the coating on the paper surface. The characteristic peaks of cellulose were clearly identified in the papers coated by lignin, GnPs and CNTs alone, but these peaks were hardly detected in the papers coated with both GnPs, CNTs and lignin, especially GM0.5-30. Since all coatings had similar thicknesses, this suggested that the mixture of GnPs, CNTs and lignin yielded more-uniform and densely packed coatings than any other combinations, which was consistent with SEM observations.

The thermal degradation of the papers coated with different carbon nanomaterials was evaluated by thermogravimetric analysis (TGA) under synthetic air, as depicted in Figure 3B. In each case, the first weight loss detected below 100 °C was associated with desorption of imbibed moisture in the papers. Interestingly, the mass decrease induced by water evaporation was much lower for papers coated with a mixture of GnPs, CNTs and lignin (i.e., ~0.5 wt %) compared to that of other samples (i.e., 5–7 wt %). Between 230 and 300 °C, all specimens experienced an abrupt reduction in mass caused by the formation of low-molecular-weight volatile chemicals due to the thermal decomposition of cellulose, which proceeded slowly above 300 °C and extended until 500 °C. The last weight loss was recorded in the temperature range of 600–700 °C due to the oxidation of nanomaterials and residual carbon. The total weight loss of blank paper was 90.5%, and the remaining 9.5% can be attributed to binders and pigments used during the paper-making process. The papers coated with either CNTs or GnPs alone yielded lower total weight losses of 88.1% and 88.7%, respectively, while the paper treated with the hybrid mixture showed the best thermal stability with a total weight loss of 84.5%. It is also worth noting that, per the manufacturer’s technical data, both GnPs and CNTs were fully oxidized above 750 °C with less than 1 wt % residual ash. Therefore, we can deduce that the residual mass of paper can be increased significantly by coating its surface with a mixture of lignin, CNTs and GnPs.

### 2.2. Gas Permeability of Coated Papers

The surface coatings can act as a protective layer to delay combustion by limiting the transfer of oxygen, decomposition gases and heat to/from the cellulose substrate underneath. Consequently, the air resistance or gas permeability of the coating played an essential role in the flame-retardant mechanism of the materials. The air resistance of the paper coated with different carbon nanomaterial combinations was evaluated by the Gurley number (Table 1). It was found that the air resistance of the papers coated with various carbon nanomaterials was significantly higher than that of the plain paper, while the gas permeability of the paper did not change after the sole deposition of lignin. This reveals that the addition of carbon nanomaterials obstructed the pores of the cellulose substrate. In addition, the papers coated with both GnPs and CNTs had a much higher air resistance compared to those coated with either component alone, indicating the superior pore-filling effect of the mixture. The paper coated with GnPs alone (i.e., G-2.5) had the lowest air resistance (i.e., 255 ± 40 s), while the paper coated with a mixture of GnPs and CNTs (i.e., GM0.5-2.5) exhibited the highest value (i.e., 903 ± 75 s), which is nearly six times larger than that of plain paper (i.e., 154 ± 3 s). These results are consistent with our SEM and FTIR characterization data. The variations in air resistance among the different types of coating can be attributed to the differences in their conformation on the cellulose substrate. Compared to one-dimensional CNTs, GnPs should theoretically show a higher air resistance due to the “tortuous path” created by their two-dimensional structure [15]. However, the degree of entanglement of two-dimensional structures is lower than their one-dimensional counterparts, and the presence of multiple micro-cracks in the sole GnP coating, as illustrated in Figure 2C, significantly hindered the air resistance of the paper. When GnPs and CNTs were mixed together, both the entangled CNT network and the “tortuous path” formed by GnPs most likely induced a synergistic effect through the creation of a physical barrier that could effectively retard the progress of gas molecules through the coated paper. The efficiency of this synergistic effect depended on the proportion of GnPs and CNTs present in the mixture. When larger quantities of GnPs were incorporated, the distance between entangled nanotubes would increase, resulting in larger size pores, thus increasing the gas permeability of the coating. Therefore, there is an optimum GnP:CNT ratio to obtain the best air resistance. Furthermore, the air resistance increased with the thickness of the coating layer, which is consistent with previous studies reporting the gas permeability of CNT membranes [33]. It was indicated that a more-compact structure was achieved by increasing the loading amount on the paper surface. Finally, the air resistance of the coated papers decreased with an increasing content of lignin, due to the lower pore-filling effect of lignin compared to that of carbon nanomaterials.

### 2.3. Flame-Retardant Performance

#### 2.3.1. Effect of Carbon Nanomaterials

**Figure 4:**
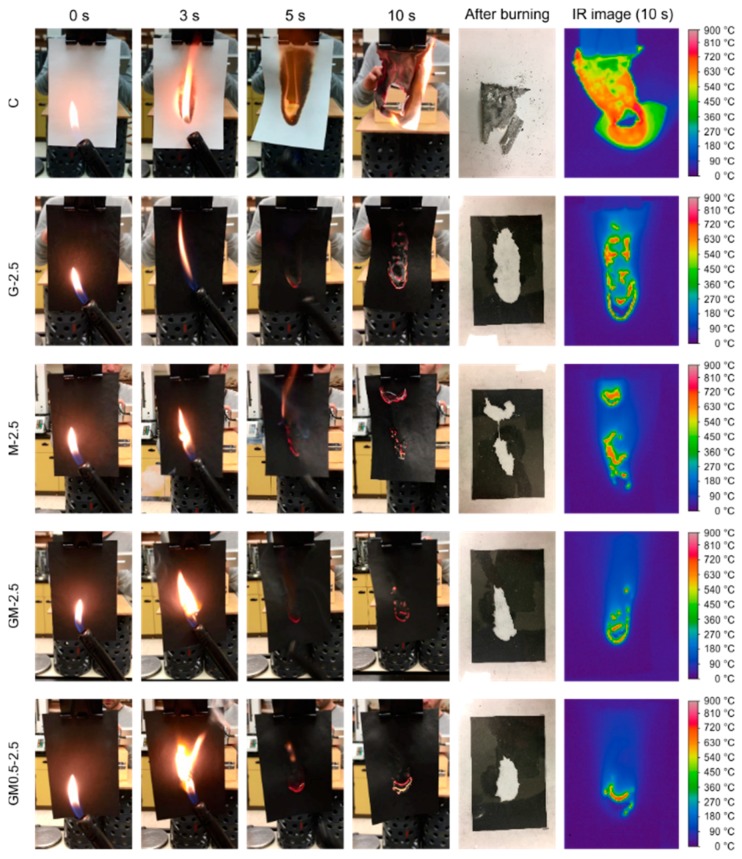
Images of the flammability test of the papers coated by different carbon nanomaterials at a loading amount of 7.5 g/m^2^ (applying the flame for 5 s), the samples with char removal after burning, and the infrared images captured by an IR camera at 10 s after contact with the flame.

A butane flame was applied to the paper surface for 5 s and then withdrawn to observe the combustion behavior (Figure 4 and Movie S1). It was seen that the plain paper kept burning after the removal of the flame until it was completely consumed in less than 15 s. In contrast, all coated papers immediately stopped burning upon the withdrawal of the flame and maintained their initial shape with little shrinkage and wrinkles, indicating excellent self-extinguishment properties. Furthermore, the combustion process varied with the coating composition. In the case of sole GnP coating (G-2.5), the papers exhibited the largest burned area, indicating the fast progression of combustion throughout the paper. For the CNT-coated papers (M-2.5), smaller burned areas were observed at multiple locations in the samples, revealing that the fire was able to spread rapidly across gaps due to locally intense heat spots. Compared to other specimens, papers coated with both GnPs and CNTs showed limited fire propagation, as illustrated by smaller burned area and the absence of flaming at locations other than that exposed to the burner. These results were consistent with IR analysis, where small independent heat spots spread over a large area of the burned paper coated with CNTs, while the heat signature of the burned paper coated with GnPs formed a large and continuous region with temperatures exceeding 450 °C, which was higher than the decomposition temperature of cellulose [37]. In the case of GnP/CNT-coated papers, fewer small heat spots were localized in the area in contact with the flame. Similar trends were observed when the flame was applied to the papers for 15 s (Figure S1 and Movie S2). To quantitatively characterize the fire-resistance properties, the burned area, weight loss and maximum temperature of the different samples were extracted from the digital images after 5 s and 15 s of contact time with a flame (Table 2). The papers coated with the GnP/CNT mixtures exhibited lower burned areas and weight losses than those coated by either component alone. In addition, the mixtures with the lowest amount of GnPs showed maximum improvements in flame-retardant characteristics compared to samples containing more GnPs, with up to 14.6% and 15.7% reductions in burned area and weight loss, respectively. Notably, the heat release was significantly reduced and the maximum temperature decreased by up to 129 °C in the presence of CNTs, which was consistent with previous studies reporting the fire resistance of polymer composites reinforced with CNTs [17,21,26].

#### 2.3.2. Effect of Coating Density

**Figure 5:**
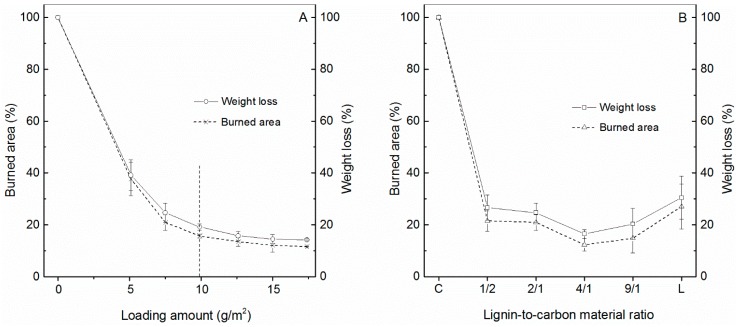
Burned areas and weight losses of the papers coated by the carbon nanomaterials with different coating density (**A**) and lignin-to-carbon material ratio (**B**). Measurements were recorded after 15 s of contact time with a butane flame.

To investigate the effect of flame-retardant concentration on the fire-resistance properties of coated papers, the loading of flame-retarding agents was varied while the coating composition was kept constant. Since it yielded the best flame-retardant properties, the coating formulation comprising a GnP:CNT mass ratio of 1:2 is reported henceforth as “GM0.5-*x*”, with *x* indicating the content of the GnP/CNT mixture in g/m^2^. From Figure S2 it is seen that all coated papers exhibited self-extinguishment properties and that the burned area became noticeably smaller as the coating density increased. The IR images also confirmed this trend with smaller and more-localized heat spots at higher coating density. Results from Figure S2 were further analyzed to determine the evolution of the weight loss and burned area as a function of coating density after 15 s of contact time with a butane flame, as depicted in Figure 5A. It was found that the burned area drastically decreased from 37.7% to 15.7% when the coating density increased from 5.1 to 9.9 g/m^2^, and then the reduction slowed down with further increments in coating density until no statistical differences in burned area were identified at 15 g/m^2^ and above. The same trend was observed in weight loss, which increased with coating density up to a certain level beyond which the additional incorporation of flame retardants did not change the weight loss of coated papers. Further addition of flame retardants above this optimal value not only represents a waste of materials but may also induce undesired effects in other substrate properties (e.g., flexibility, optical density, cost and total mass), which is consistent with a previous study reporting a similar phenomenon in the case of polymer nanocomposites reinforced with graphene oxide [38].

#### 2.3.3. Effect of the Ratio of Lignin to Carbon Nanomaterials

The effect of lignin content on the combustion behavior of the coated papers was investigated by varying the mass ratio of lignin to carbon nanomaterials from 1:2 to 9:1. In each case, the carbon nanomaterial composition was kept constant with a GnP:CNT mass ratio of 1:2, and the coating density was maintained at 7.5 g/m^2^. The samples were designated as LGM*x-y*, with *x* indicating the ratio of lignin to carbon nanomaterials and *y* indicating the content of GnPs and CNTs. From Figure S3 it is observed that the coating paper containing carbon nanomaterials maintained the self-extinguishing properties regardless of the lignin concentration, while the paper coated by solely lignin kept burning after the flame was withdrawn. Moreover, the calculated burned area and weight loss decreased with the addition of lignin up to a certain level, beyond which further increase in lignin concentration led to a slight augmentation of the burned area and weight loss (Figure 5B). The lowest values of burned area (i.e., 12.3%) and weight loss (i.e., 16.5%) were achieved with a lignin-to-carbon nanomaterial ratio of 4:1. Interestingly, these values were equivalent or even lower than the burned area and weight loss reported at higher coating densities. This means that the coating density can be significantly reduced while maintaining the same level of flame-retardant properties by adjusting the lignin-to-carbon nanomaterial ratio, providing great opportunities for cost reduction. In this study, the total mass content of carbon nanomaterials was as low as 4 wt %.

## 3. Flame-Retardant Mechanism

**Figure 6:**
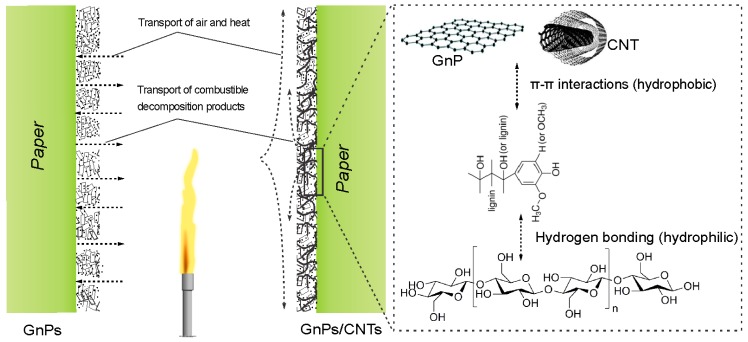
The illustration demonstrating the flame-retardant mechanism of GnP/CNT/lignin-coated cellulosic papers.

The superior flame-resistance properties of the coated papers at low flame-retardant content can be attributed to the synergistic effects between GnPs, CNTs and lignin, improving (i) the physical barrier characteristics, (ii) the formation of thermally stable char and (iii) the thermal management of the system. First, the presence of a physical barrier between the substrate and the environment is critical to limit the transport of oxygen and other gaseous products during combustion. The combination between one dimensionalCNTs and two dimensional GnPs has closed the gaps between them (Figure 1) and formed a denser coating layer on the paper surface with reduced gas permeability (Table 1). The lower porosity of this hybrid structure and the presence of a “tortuous path” induced by the evenly distributed GnPs in the CNT network were able to greatly hinder mass and heat transfers between the paper and the flame. However, simply mixing CNTs and GnPs was not sufficient to create an efficient physical barrier, and the dispersion state of these carbon nanomaterials is of paramount importance. Previous research reported that the flame-retardant properties of polymer composites with poorly dispersed CNTs did not improve compared to the pristine composites without CNTs [39]. Owing to its large number of condensed aromatic structures, lignin was adsorbed on GnPs and CNTs via π-π interactions and prevented the nanomaterials from sticking together due to electrostatic repulsion and steric hindrance during the aqueous-phase coating process, as demonstrated elsewhere [28,32]. Moreover, multiple hydrophilic groups in the lignin structure (e.g., methoxyl, phenolic hydroxyl, aldehyde and carboxyl) can interact with cellulose via hydrogen bonding, hence connecting the carbon nanomaterials to the paper substrate. Therefore, the role of lignin is twofold: (i) it promotes the aqueous dispersion of carbon nanomaterials [28], and (ii) serves as a cross-linking agent to bridge the cellulose microfibers with carbon nanomaterials (Figure 6). This results in the formation of a compact layer protecting the substrate from the flame and preserving the paper integrity, which will also be responsible for the self-extinguishing properties. Without the combination of all these elements, the coating appeared to be cracked (i.e., G-2.5), with open pores of several hundred nanometers in size (i.e., M-2.5), and showed high gas permeability (i.e., L), which was detrimental to the fire resistance of the papers. Furthermore, when the concentration of lignin in the coating increased up to a certain amount, the flame-retardant properties of the papers were improved, while the gas permeability increased. This suggests that a different flame-retardant mechanism occurs at higher lignin content. Lignin is known as a char-forming agent and its lower degradation temperature compared to that of carbon nanomaterials will facilitate the formation of carbonaceous residue during combustion, which can balance, to some extent, the augmentation of gas permeability at high lignin content. This char can further shield the substrate from the flame by filling in voids between adjacent particles, serving as a thermal insulating barrier inhibiting mass and heat transfers between the paper and its surroundings. Finally, thermal management was also greatly improved by the different carbon materials in the coating. CNTs were efficient heat sinks that can absorb heat from the ignition source, while GnPs can diffuse the heat energy away from the source [40]. When used separately, CNTs could cool the paper surface down from 831 °C to 709 °C (Table 2) but generated local and intense hot spots causing additional flaming areas, while GnPs spread the heat to wider areas, speeding up the combustion process. Their combination, on the other hand, can concurrently cool down the surface, delay combustion and prevent flame propagation. In the hybrid coating, the heat energy is rapidly transferred from the GnPs to the CNTs, where the existence of heat-sink regions ensures efficient heat dissipation [41]. In addition, adjacent GnPs and CNTs will provide an increased number of direct contacts compared to either component alone (Figure 1), closing the gaps between them and forming a denser hierarchical network with larger numbers of thermally conductive pathways for better thermal management.

## 4. Materials and Methods

### 4.1. Materials

Pristine copy papers with an average density of 0.65 g/cm^3^ were obtained from North Pacific Paper Company (Longview, WA, USA). GnPs with an average surface area of 750 m^2^/g were supplied by Sigma-Aldrich Corp. (St. Louis, MO, USA). Multi-wall CNTs with length of 10–20 μm and mean outer diameter of 50 nm synthesized by catalytic chemical vapor deposition and purified using acid chemistry were provided by Cheap Tubes Inc. (Grafton, VT, USA). The alkali lignin was purchased from Tokyo Chemical Industry Co. (Tokyo, Japan). Deionized (DI) water served as solvent for the nanomaterial dispersions. All chemicals were used as received without any further treatment.

### 4.2. Preparation of GnP/CNT/Lignin-Coated Paper

The preparation of flame-retardant papers is summarized in Figure 1. A certain amount of alkali lignin was dispersed in DI water and stirred for 10 min. Then, the lignin dispersion was bath-sonicated for 10 min by an ultrasonicator (VWR International, LLC, Radnor, PA, USA). A desired amount of GnPs and/or CNTs was added into the suspension and double sonicated based on a previously established procedure [28]. This method allows for the preparation of stable and concentrated suspensions of individualized nanomaterials with significantly reduced processing time and improved dispersion quality. The different coating formulations are listed in Table 1. Subsequently, the dispersions were applied onto both sides of the plain paper by Meyer rod coating in a single deposition step. A smooth rod moving at a speed of 150 mm/s was used to enable the production of large and uniform films in short times. The rod moving speed was not a critical factor and could be slightly adjusted without affecting coating quality [42]. The coated samples were air dried and conditioned under a controlled atmosphere (i.e., 50% humidity, 20 °C) for at least 48 h prior to further characterization.

### 4.3. Characterization

Surface morphologies of the GnP/CNT-coated paper were examined by a scanning electron microscope (SEM, XL830, FEI Company, Hillsboro, OR, USA) operated at 5 kV. The plain paper without coating was sputtered with a thin layer of gold prior to SEM observation. The chemical structure information of the samples was obtained by a Shimadzu Fourier transform infrared (FTIR) spectrophotometer with an attenuated total reflectance (ATR) mode (IRPrestige-21, Shimadzu Corp., Kyoto, Japan). All spectra were collected within the wavenumber range of 600–4000 cm^−1^ at a spectral resolution of 4 cm^−1^. Thickness was measured at 15 different spots per sample using a Electronic Thickness Tester Model 2 (Thwing-Albert Instrument Co., West Berlin, NJ, USA) operated at 7.3 psi in accordance with the Technical Association of the Pulp and Paper Industry standard method (TAPPI, T411) to ensure repeatability of the thickness values. Thermogravimetric analysis (TGA) was performed on a NETZSCH STA-449-F3 (Selb, Germany). About 25 mg of sample was placed into a platinium-rhodium alloycrucible and heated from room temperature to 850 °C at a rate of 10 °C/min under a synthetic air atmosphere (i.e., oxygen at 25 mL/min and nitrogen at 25 mL/min). The gas permeability of the papers was measured to evaluate the barrier effect of the coating materials based on the Gurley method (TAPPI, T460 cm-88). The Gurley number, which can be directly correlated to the air resistance of the material [43], was defined as the time for a given volume (100 mL) of air to flow through 1 in^2^ (6.45 cm^2^) circular area of paper under constant pressure by the test apparatus (Model 4110, Gurley Precision Instruments, Troy, NY, USA). At least three measurements were performed to obtain the average air resistance value of each sample.

### 4.4. Flame Resistance Test

The vertical flame tests were carried out in triplicate based on the TAPPI, T461 cm-00 standard method. Briefly, a suspended 60 mm × 90 mm paper was exposed to a butane flame in an atmosphere in accordance with TAPPI T402 (Figure 1). The flame of the burner was adjusted to a height of 40 mm and applied to the center of the specimen for 15 s (i.e., the flame itself was touching the sample surface). Shorter flammability tests (i.e., at 5 s ignition times) were also conducted to analyze the materials’ self-extinguishing properties. The burning evolution of the papers was captured by a video camera. After it ceased to flame, the sample was removed from the holder and the charred area was gently tapped to break away loose char. The weight loss (W_L_) was recorded using a Mettler Toledo AG285 analytical balance (Columbus, OH, USA) and the burned area (A_L_) was determined using ImageJ based on the following equations,

W_L_ = 100 ∗ (W_i_ − W_f_)/W_i_ (1)

A_L_ = 100 ∗ (A_i_ − A_f_)/A_f_, (2)

where W_L_, W_i_, W_f_, A_L_, A_i_ and A_f_ are the weight loss, initial weight, final weight after burning, burned area, initial area, and remaining area after burning of the tested paper, respectively. Furthermore, an IR camera equipped with a pyrometer (MikroScan 7600, LumaSense Technologies, Inc., Santa Clara, CA, USA) was used to monitor the temperature distribution of the sample while burning. The emissivity was set to 0.90 and 0.96 for the pristine and coated papers, respectively. All flame resistance experiments were performed three times and average values of W_L_, A_L_ and maximum temperature were calculated.

## 5. Conclusions

Cellulosic paper was endowed with extraordinary self-extinguishing properties and flame retardancy by coating its surface with GnPs, CNTs and lignin using a Meyer rod-coating method. Carbon nanomaterials pre-adsorbed with alkali lignin created a hierarchical network structure forming a compact layer on the paper surface, which served as a protective film during combustion. Such a carbonaceous shield was able to retard greatly the combustion progress of the material by reducing the transport of air and decomposition byproducts from/to the substrate. Papers coated by combinations of GnPs, CNTs and lignin exhibited a more-uniform and compact structure with lower air permeability and higher amounts of thermally conductive pathways resulting in enhanced flame-retardant performance compared to papers coated by either component alone. Moreover, the fire-resistance properties gradually reached a maximum after a certain quantity of flame retardant was applied to the paper surfaces, and the lignin content played an essential role as char-forming agent. Using a tailored coating of unmodified commercially available carbon nanomaterials with specific composition and density, we were able to achieve superior fire resistance properties, while keeping the amount of carbon nanomaterials to a minimum level. We expect that these findings will guide future optimization of advanced and affordable flame-retarding agents for minimizing fire risk, meeting safety regulations and expanding high-value application of cellulose and other polymer materials.

## Figures and Tables

**Table 1:** Formulation of the coating materials on the papers and air resistance of the treated papers.

Sample	GnPs (mg/mL)	CNTs (mg/mL)	Lignin (mg/mL)	Coating Density w/–w/o Lignin (g/m^2^)	Thickness (μm)	Gurley Number (s/100 mL)
C *	0	0	0	0–0	113 ± 6	154 ± 3
G-2.5 *	10	0	20	7.5–2.5	168 ± 6	255 ± 40
M-2.5 *	0	10	20	7.5–2.5	164 ± 3	765 ± 108
GM-2.5 *	5	5	20	7.5–2.5	165 ± 5	889 ± 119
GM0.5-2.5 *	3.3	6.7	20	7.5–2.5	169 ± 8	903 ± 75
GM0.5-1.7 *	3.3	6.7	20	5.1–1.7	166 ± 3	411 ± 47
GM0.5-3.3 *	3.3	6.7	20	9.9–3.3	176 ± 16	1199 ± 262
GM0.5-4.2 *	3.3	6.7	20	12.6–4.2	180 ± 10	1307 ± 299
GM0.5-5.0 *	3.3	6.7	20	15.0–5.0	184 ± 20	1661 ± 172
GM0.5-5.8 *	3.3	6.7	20	17.4–5.8	188 ± 10	2368 ± 334
LGM0.5-5.0 *	6.7	13.3	10	7.5–5.0	192 ± 13	1717 ± 240
LGM2-2.5 *	3.3	6.7	20	7.5–2.5	169 ± 8	903 ± 75
LGM4-1.5 *	2	4	24	7.5–1.5	167 ± 9	637 ± 53
LGM9-0.75 *	1	2	27	7.5–0.75	168 ± 6	423 ± 53
L *	0	0	30	7.5–0	163 ± 5	202 ± 12

* Sample designation is as follows: G, M and L refer to papers coated with graphene nanoplatelets (GnPs), carbon nanotubes (CNTs) and lignin (and their corresponding mixtures), respectively. C designates the plain paper used as control experiment. For heterogeneous coatings, the first number following the type of material indicates the mass ratio of the mixture. For instance, GM0.5 corresponds to a GnP:CNT ratio of 1:2, while LGM4 refer to a lignin:GnP/CNT ratio of 4:1. The last number indicates the coating density of carbon nanomaterials (i.e., GnPs and CNTs) in g/m^2^ excluding the lignin.

**Table 2:** The burned area, weight loss and maximum temperature after the flame-retardant testing of the papers burned for 5 s and 15 s.

Sample	Burned Area (%)		Weight Loss (%)		Maximum Temperature (°C)	
-	5 s	15 s	5 s	15 s	5 s	15 s
C	100	100	100	100	809 ± 71	831 ± 53
G-2.5	15.7 ± 3.4	35.5 ± 1.5	18.2 ± 6.0	40.4 ± 2.7	791 ± 11	834 ± 42
M-2.5	12.4 ± 2.7	21.0 ± 2.6	16.05 ± 1.8	25.3 ± 2.9	766 ± 5	705 ± 13
GM-2.5	12.0 ± 3.1	22.5 ± 2.5	14.6 ± 2.9	26.4 ± 1.5	760 ± 3	709 ± 3
GM-0.5-2.5	10.7 ± 2.9	20.9 ± 3.0	13.5 ± 2.7	24.7 ± 3.8	749 ± 16	730 ± 23

## Supplementary Materials

Supplementary materials can be found at www.mdpi.com/1422-0067/18/11/2368/s1.
