# Supplementary material for: Lignin-Modified Carbon Nanotube/Graphene Hybrid Coating as Efficient Flame Retardant
Source: Int J Mol Sci. 2017 Nov 8;18(11):2368. doi: 10.3390/ijms18112368 (PMC5713337; doi:10.3390/ijms18112368)
Supplement: Supplementary file 1 [file ijms-18-02368-s001.zip › ijms-239106-supplementary materials.pdf]

Supporting Information

Lignin-modified carbon nanotube/graphene hybrid coating as efficient flame retardant

Kunlin Song[1], Indroneil Ganguly[2], Ivan Eastin[2], Anthony B. Dichiara[1]*

[1]School of Environmental and Forest Sciences, University of Washington, Seattle, WA 98195, USA

[2]CINTRAFOR, School of Environmental and Forest Sciences, University of Washington, Seattle, WA 98195, USA

*Corresponding author: Tel: (206)543-1581. E-mail: abdichia@uw.edu

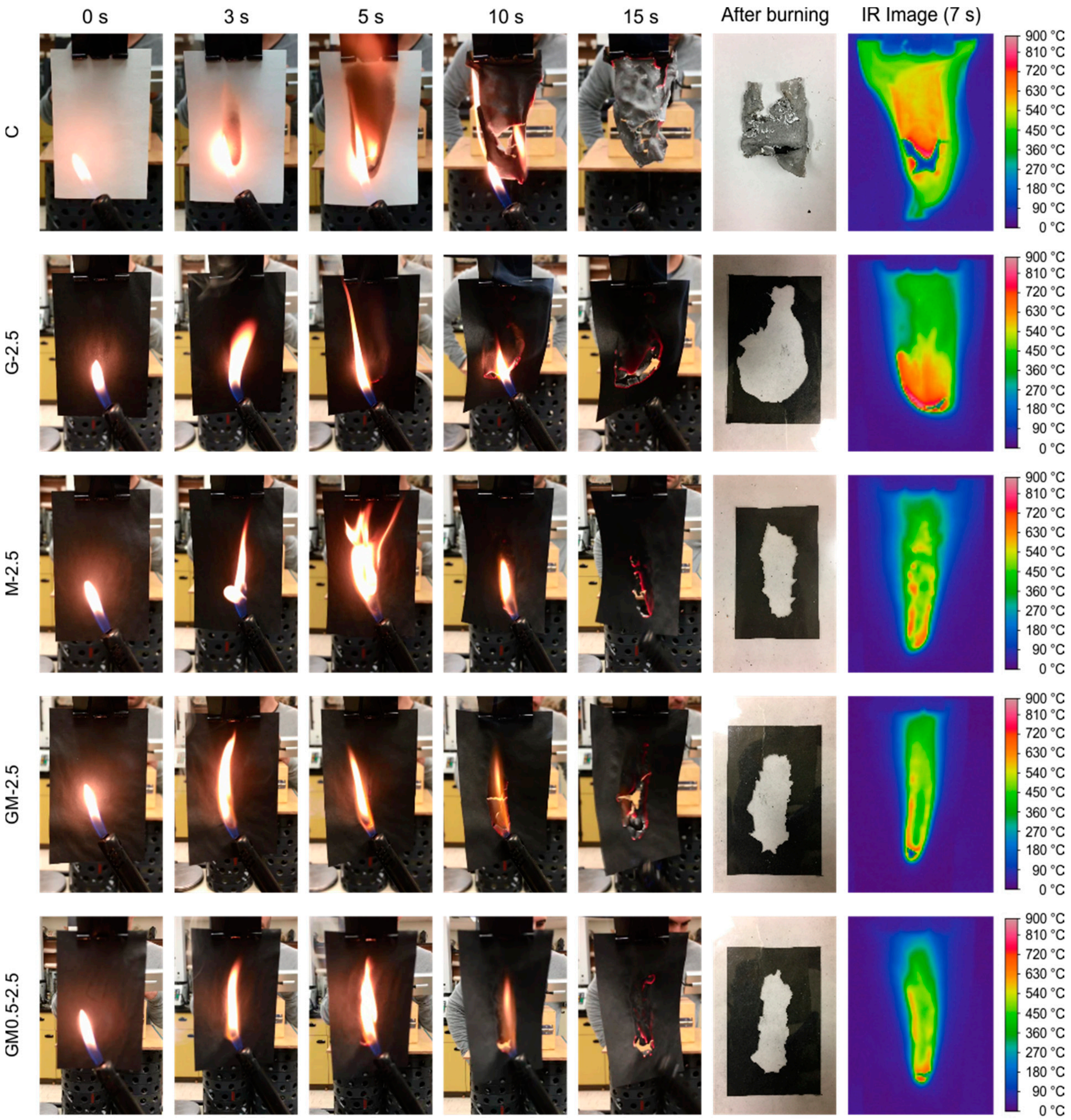

Figure S1. Images of the flammability test of papers coated with different carbon nanomaterials. The flame was applied for 15 s. Photographs of the samples after char removal and thermal images captured by an IR camera after 7 s of combustion are also depicted.

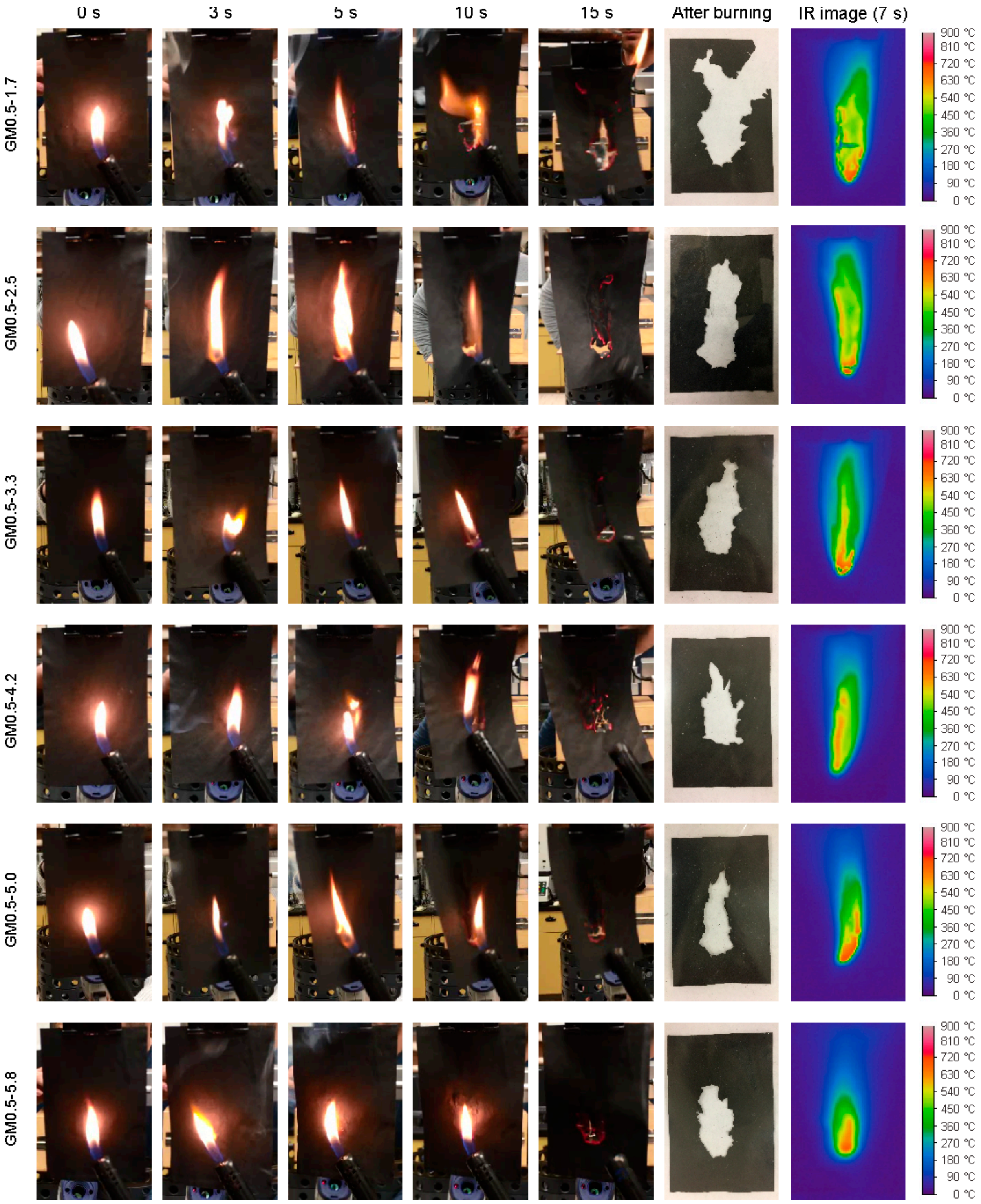

Figure S2. Images of the flame retardant testing of the papers coated with different densities of GnP/CNT mixtures. The flame was applied for 15 s. Photographs of the samples after char removal and thermal images captured by an IR camera after 7 s of combustion are also depicted.

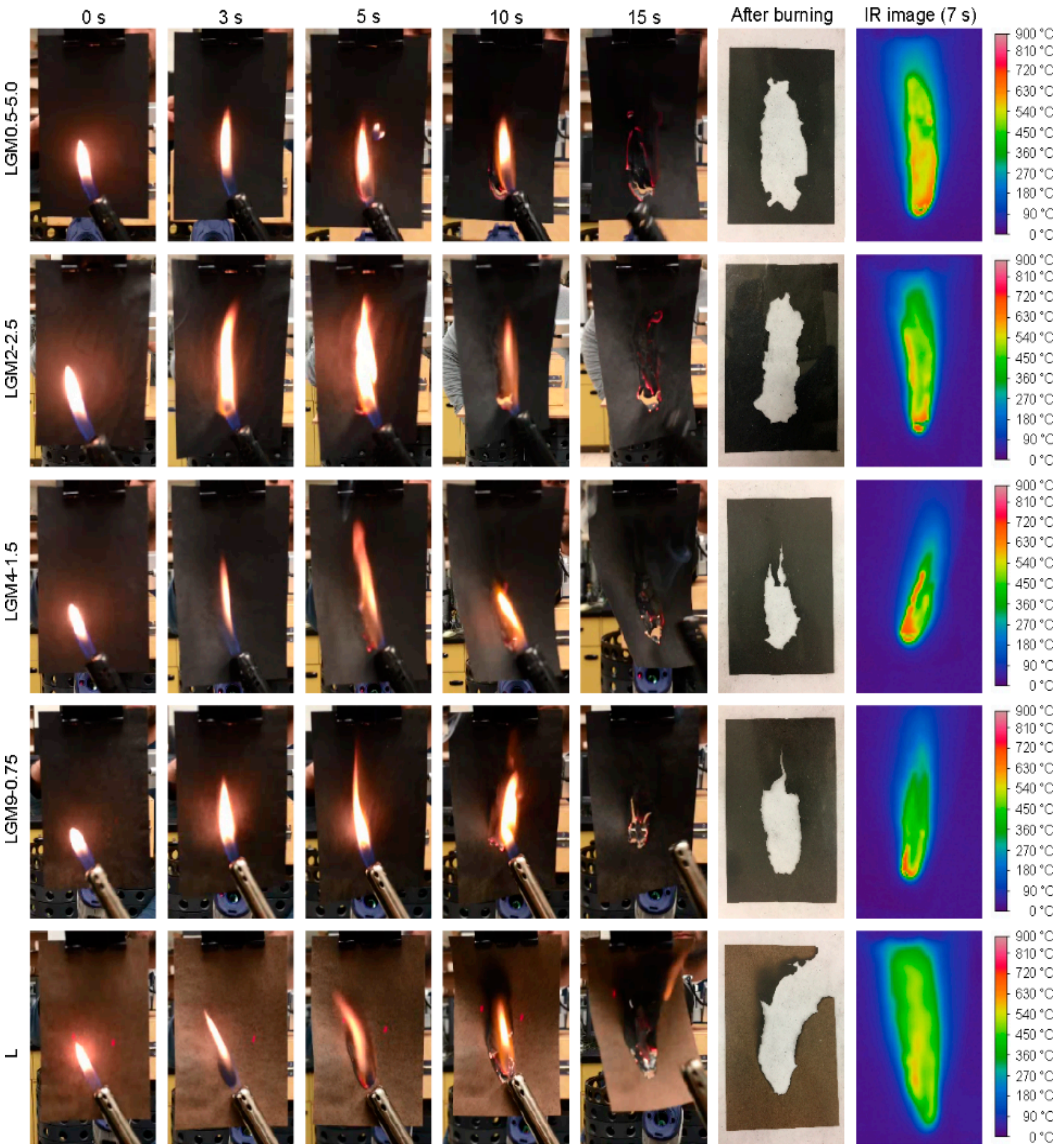

Figure S3. Images of the flame retardant testing of the coated papers with different lignin to carbon nanomaterials ratios. The butane flame was applied for 15 s. Photographs of the samples after char removal and thermal images captured by an IR camera after 7 s of combustion are also depicted.

Movie S1. Video of the 5 s flammability test for the plain and GnP/CNT coated papers.

Movie S2. Video of the 15 s flammability test for the plain and GnP/CNT coated papers.
